# *Adetogramma* (Polypodiaceae), a new monotypic fern genus segregated from Polypodium

**DOI:** 10.3897/phytokeys.78.12189

**Published:** 2017-04-13

**Authors:** Thaís Elias Almeida, Alexandre Salino, Jean-Yves Dubuisson, Sabine Hennequin

**Affiliations:** 1 Universidade Federal do Oeste do Pará – Herbário HSTM, Avenida Marechal Rondon, s.n. – Santarém, Pará, Brazil. CEP: 68040-070; 2 Programa de Pós-graduação em Biologia Vegetal - Departamento de Botânica, Universidade Federal de Minas Gerais, Av. Antônio Carlos, 6627 – Belo Horizonte, Minas Gerais, Brazil. Caixa Postal 486, CEP 31270-901; 3 Institut Systématique Evolution Biodiversité (ISYEB), Sorbonne Universités, UPMC Univ. Paris 06, MNHN, CNRS, EPHE, 75005 Paris, France; 4 Centre de Recherche en Paléobodiversité et Paléoenvironnements (CR2P), Sorbonne Universités, UPMC Univ. Paris 06, MNHN, CNRS, 75005 Paris, France

**Keywords:** Andes, Polypodiaceae, phylogeny, *Serpocaulon*, taxonomy

## Abstract

Polypodiaceae is one of the most diverse and abundant families of ferns in tropical and subtropical forests. Despite multiple studies investigating its phylogeny and taxonomy, several generic boundaries within the family still need clarification. One of the most problematic circumscriptions is that of *Polypodium* L., and one species that still contributes to this uncertainty is *Polypodium chrysolepis* Hook. The main goal of this study was to use molecular and morphological data to clarify the relationships of *P. chrysolepis* inside the polygrammoid clade. Sequences from three plastid regions (cpDNA – *rbc*L, *rps*4 and *rps*4–*trn*S IGS) from fifty species belonging to thirty-two genera of Polypodiaceae were analyzed using maximum likelihood and Bayesian inference. *Polypodium chrysolepis* constitutes an isolated lineage among the neotropical polygrammoid ferns, close to *Serpocaulon* and the grammitids, and is recognized here in a new genus. It can be distinguished by its entire leaves with free veins and peltate, pedicellate, lanceolate paraphyses. A new combination, *Adetogramma chrysolepis*, is proposed and a new taxonomic treatment is presented; its conservation status was assessed using IUCN Red List Categories and Criteria.

## Introduction

Polypodiaceae is one of the richest fern families, and one of the most diverse and abundant groups of vascular plants in tropical and subtropical forests (Schneider et al. 2004). The current family circumscription (PPG I 2016) is based on many phylogenetic studies (e.g., Schneider et al. 2004, Schuettpelz and Pryer 2007). With this definition, Polypodiaceae includes the large segregate family, Grammitidaceae (sensu Parris 1990), and comprises 65 genera (PPG I 2016). Despite multiple studies investigating the phylogeny and taxonomy of Polypodiaceae, Smith et al. (2008) recognized that certain generic boundaries within the family still need clarification.

One of the most problematic circumscriptions is that of *Polypodium* L. (Smith et al. 2008). As recognized by Hennipman et al. (1990), *Polypodium* is polyphyletic, and several groups historically included in it (Hennipman et al. 1990) have been recognized as other genera [e.g., *Goniophlebium* (Blume) C.Presl, *Microgramma* C.Presl (Salino et al. 2008), *Pleopeltis* Willd., *Phlebodium* (R.Br.) J.Sm. (Otto et al. 2009), *Serpocaulon* A.R.Sm. (Smith et al. 2006a), *Synammia* C.Presl (Schneider et al. 2006)]. Nevertheless, recognition of all the above-cited genera still does not render *Polypodium* monophyletic (Schneider et al. 2004, Otto et al. 2009, Assis et al. 2016). In its latest circumscription (PPG I 2016), the genus is considered presumably monophyletic, but the groups that remain in it [*Polypodium dulce* group, *Polypodium plesiosorum* group, and *Polypodium colpodes* group (Tejero-Díez 2005, Sigel et al. 2014)], still need to be comprehensively included in phylogenetic studies.

One species that still contributes to this uncertainty is *Polypodium chrysolepis* Hook., a species occurring in the Andes from northern Argentina to Ecuador. The generic placement of this species has been controversial: it was described in *Polypodium* by Hooker (1844) in *Icones Plantarum*, tentatively placed in *Lepicystis* [now treated within *Pleopeltis* (Smith and Tejero-Díez 2014)] by Diels (1899), recognized as a distinct entity within *Polypodium* by de la Sota (1960) and placed in *Microgramma* by Crabbe (1967), following notes by A.H.G. Alston. The first molecular phylogenetic placement of *P. chrysolepis* (Schneider et al. 2004) showed that none of the above-mentioned generic positions are acceptable. In Schneider et al. (2004), *P. chrysolepis* was recovered as sister to the *Serpocaulon*+grammitids clade, and it is distantly related to the campyloneuroid clade (which includes *Campyloneurum*, *Microgramma*, and *Niphidium*) and to *Polypodium* *s.s.* clade. No formal taxonomic changes were proposed for *P. chrysolepis* by Schneider et al. (2004), as its position was ambiguous, and since then no new studies have been conducted on this species.

The main goal of this study was to employ molecular and morphological data to investigate the relationships of *Polypodium chrysolepis* within the polygrammoid clade, and to use available morphological and phylogenetic information to formally propose an adequate generic placement for this species in Polypodiaceae.

## Material and methods

### Taxon sampling

Fifty species from thirty-two genera (sensu PPG I 2016) of Polypodiaceae were included in our phylogenetic analyses (Appendix). *Davallia solida* (G.Forst.) Sw. (Davalliaceae) and *Oleandra pistillaris* (Sw.) C.Chr. (Oleandraceae) were used as outgroups, following Schneider et al. (2004). All vouchers and GenBank accessions are listed in the Appendix. Aligned data matrix was deposited in TreeBASE (http://purl.org/phylo/treebase/phylows/study/TB2:S20420).

### Sequence acquisition

Total DNA was extracted from field-acquired silica gel-dried or fresh tissues, using the Qiagen DNeasy Plant mini kit (Qiagen Inc., Valencia, CA, USA). PCR amplifications were performed for two chloroplast genome regions: *rbc*L (coding region; ca. 1,300 bp) and *rps*4 (the coding region *rps*4 and the intergenic spacer *rps*4–*trn*S; ca. 1,100 bp). Amplifications were done in a single reaction with primers 1F and 1365R for the *rbc*L region (Haufler and Ranker 1995) and primers rps5F (Nadot et al. 1995) and trnSR (Smith and Cranfill 2002) for the *rps*4 and *rps*4-*trn*S regions. These regions have shown their utility for inferring phylogenetic relationships in Polypodiaceae, as shown in Janssen and Schneider (2005), Janssen et al. (2007), Kreier and Schneider (2006), Kreier et al. (2007), Kreier et al. (2008), Schneider et al. (2002), Schneider et al. (2004), and Salino et al. (2008).

Polymerase chain reactions were performed in a 20 μL solution containing 1.0 μL of non-diluted genomic DNA template, 2.0 μL of PCR buffer (Qiagen 10 × PCR Buffer), 1.0 μL of DMSO, 1.0 μL of BSA (4 mg/mL), 0.8 μL of dNTPS (10 mM), 0.32 μL (10 μM) of each primer, 0.12 units of *Taq* Dna polymerase (Qiagen, 5 units μL), and 14.44 μL of ultra-pure water. Thermal cycling conditions were the same for both regions: 3 min at 94°C, 35 cycles of 45 s at 94°C, 60 s at 53°C and 90 s at 72°C, followed by 5 min at 72°C. Amplicons were purified by precipitation with polyethylene glycol (PEG) and sequenced by Macrogen (Seoul, South Korea) in a bidirectional sequencing reaction in an ABI3730XL.

### Alignment and phylogenetic analyses

Sequence electropherograms were edited using the STADEN package software (Bonfield et al. 1995). Edited sequences were submitted to automated alignment with MUSCLE (Edgar 2004) and the resulting alignment was checked manually using MEGA 7 (Kumar et al. 2016).

Datasets were analyzed using maximum likelihood (ML) and Bayesian inference (BI). Maximum likelihood was performed using IQ-TREE (Nguyen et al. 2015) with partitioned matrix (Chernomor et al. 2016), automatic selection of the best-fit substitution model (using Bayesian Information Criterion, Schwarz 1978), and branch support assessed with 10,000 ultrafast bootstrap replicates (Minh et al. 2013). Best-fit models according to BIC were TIM2e+G4 for *rbc*L, K3Pu+G4 for *rps*4 gene and TVM+G4 for *rps*4-*trn*S IGS. For BI, a model-based phylogenetic analysis using Markov chain Monte Carlo-based was performed using MrBayes v3.2.2 (Ronquist et al. 2012), treating each DNA region (*rbc*L, *rps*4 gene and *rps*4-*trn*S IGS) as separate partitions. An evolutionary model for each DNA region was selected in jModelTest 2 (Darriba et al. 2012; Guindon and Gascuel 2003), using the Bayesian Information Criterion (Schwarz 1978, Table 1). For the *rbc*L dataset, the SYM+I+G was selected, and for the *rps*4 gene and *rps*4-*trn*S datasets the GTR+G model was selected. Each analysis consisted of two independent runs with four simultaneous Markov Chains run for 5,000,000 metropolis-coupled generations, starting with random initial trees and sampling one tree every 1000 generations. To improve swapping of chains the temperature parameter for heating the chains was lowered to 0.05. To check the convergence of the runs, ESS (effective sample size) and PSRF (potential scale reduction factor) were examined (Ronquist et al. 2012) using Tracer v.1.6 (Rambaut et al. 2014). Based on the sampled parameter values examined, 2000 trees, including the ones generated during the burn-in phase, were discarded. Remaining trees were used to assess topology and posterior probabilities (PP) in a majority-rule consensus tree. Because PP in Bayesian analysis are not equivalent to bootstrap (BP) (Erixon et al. 2003), we used criteria similar to a standard statistical test, considering groups with PP > 95% as strongly supported, PP 90–95% as moderately supported and PP < 90% as weakly supported. Results were summarized on a majority rule consensus tree.

**Table 1.** Selected models and parameter values for data partitions used in this study.

Region	Base frequencies								
	Selected model	A	C	G	T				
*rps*4 gene	GTR+G	0.3204	0.1924	0.1964	0.2908				
*rsp*4-*trn*S IGS	GTR+G	0.3249	0.1545	0.1605	0.3601				
*rbc*L	SYM+I+G	–	–	–	–				
	**Substitution model (rate matrix)**								
	**A-C**	**A-G**	**A-T**	**C-G**	**C-T**	**G-T**	**Ti/tv**	**I**	**G**
*rps*4 gene	0.7965	3.5099	0.1724	0.4720	3.0330	1	0	0	0.9640
*rsp*4-*trn*S IGS	0.9825	2.7675	0.2147	0.7566	2.9398	1	0	0	0.5615
*rbc*L	1.9337	8.1010	1.1861	0.8284	11.3400	1	0.5474	0	0

### Taxonomic studies and conservation status

Taxonomic conclusions were based on the study of specimens from the following herbaria: BHCB, BM, BR, G, GH, K, LPB, M, MO, NY, P, Q, QCA, QCNE, QPLS, US, USM, and USZ. Specimens with barcode are cited with herbarium acronym followed by barcode number. Abbreviation of genera and species followed IPNI (ipni.org) and morphological terms follow Lellinger (2002). Scanning electron microscope (SEM) images were made using a FEI Quanta 200 SEM, with an accelerating voltage of 30 kV. Samples were sputter-coated with gold and imaged digitally. Spore terminology follows Tryon and Lugardon (1991). Conservation status was assessed using IUCN Red List Categories and Criteria (IUCN 2016) to calculate the Extent of Occurrence (EOO) and the Area of Occupancy (AOO), using the GeoCAT tool (Bachman et al. 2011). The specimens that did not present coordinates were attributed one, whenever locality information was available. A grid cell of 10 km^2^ was used for AOO estimation.

## Results

**Figure 1.**
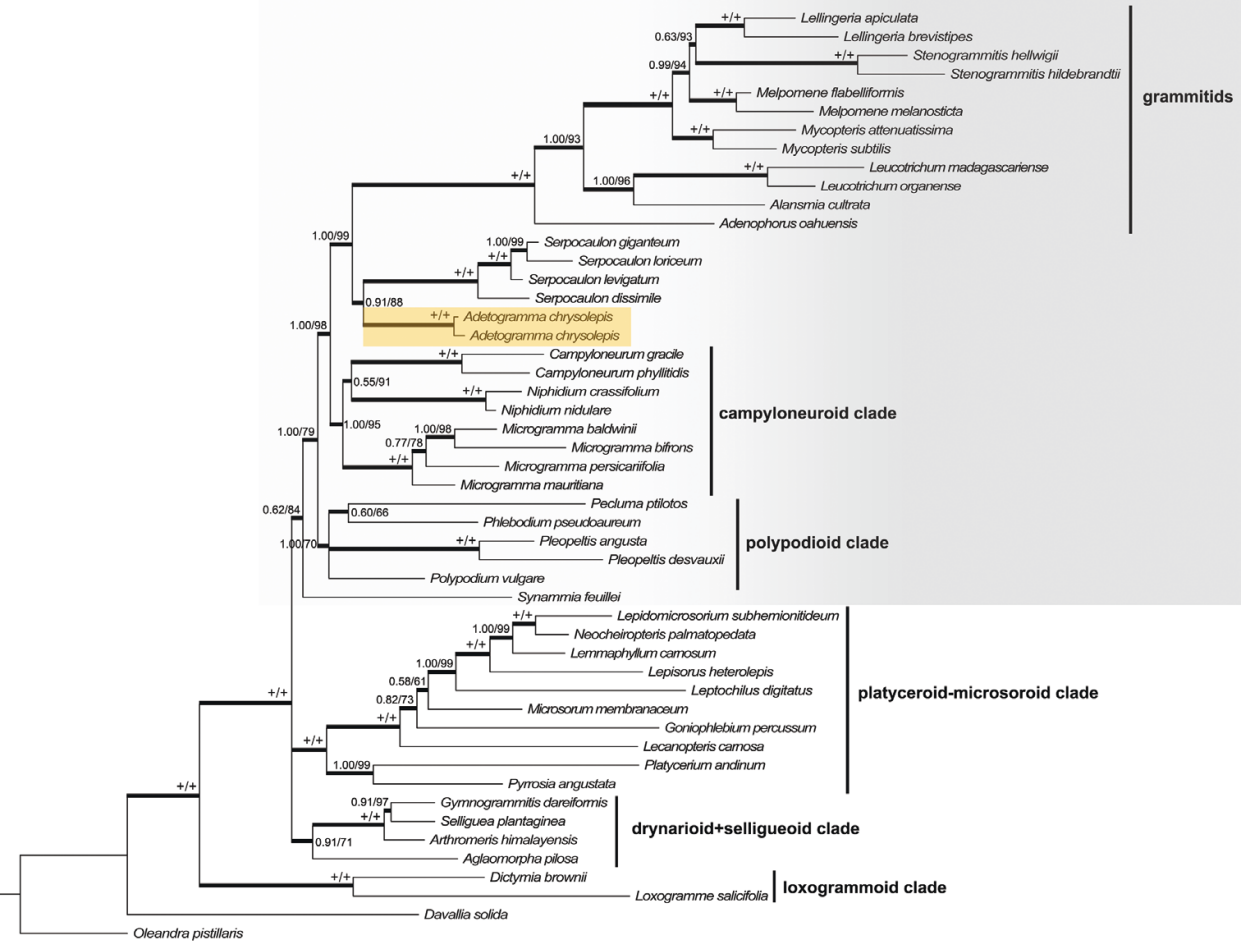
Majority rule consensus tree resultant from Bayesian inference analyses from combined *rbc*L and *rps*4 datasets. Values above branches nodes correspond to Bayesian posterior probabilities and maximum likelihood bootstrap support, respectively. +/+ indicates with 1.00 PP and 100% BS support. Clades cited in text follow Schneider et al. (2004); gray shade indicates neotropical Polypodiaceae. *Adetogramma chrysolepis* is highlighted in orange.

The final combined dataset presented 2339 bp, 599 from *rps*4 gene, 473 from the *rps*4-*trn*S IGS, and 1267 from *rbc*L. All analyses recovered the main polygrammoid clades found by Schneider et al. (2004; see Fig. 1): the loxogrammoid clade (1.00 PP, 100% BS), the drynarioid + selligueoid clade (0.91 PP, 71% BS), the platyceroid-microsoroid clade (1.00 PP, 100% BS), and a clade comprising neotropical representatives (0.62 PP, 84% BS).

Inside the neotropical clade, *Synammia* appears as sister to all the other neotropical clades, a result also obtained by Schneider et al. (2006) (Fig. 1). Sister to *Synammia*, our analyses recovered three main clades: the polypodioid clade including *Pecluma*, *Phlebodium*, *Pleopeltis*, and *Polypodium* *s.s.* (1.00 PP, 70% BS,), the campyloneuroid clade, containing *Campyloneurum*, *Microgramma*, and *Niphidium* (1.00 PP, 95% BS), and a clade (1.00 PP, 99% BS) that included the grammitid ferns (1.00 PP, 100% BS), *Serpocaulon* (1.00 PP, 100% BS), and *Polypodium chrysolepis* (1.00 PP, 100% BS). Both maximum likelihood and Bayesian inference hypotheses recovered *P. chrysolepis* as sister to *Serpocaulon* (0.91 PP, 88% BS).

## Discussion

The polygrammoid topology recovered in our analyses agrees with previous results from several studies (Schneider et al. 2004, Schneider et al. 2006, Smith et al. 2006b, Salino et al. 2008, Otto et al. 2009). The placement of *Polypodium chrysolepis* as an isolated lineage inside neotropical Polypodiaceae and closely related to *Serpocaulon* and the grammitid ferns (1.00 PP, 97% BS) confirms results previously found by Schneider et al. (2004); this species is not part of the campyloneuroid clade, containing *Microgramma*, nor does it belong to the polypodioid clade, containing *Polypodium* *s.s.* and *Pleopeltis*. The afore-mentioned genera have all been used as a “home” for *Polypodium chrysolepis* by various authors in the past (e.g., Hooker 1844, de la Sota 1960, Crabbe 1967).

**Figure 2.**
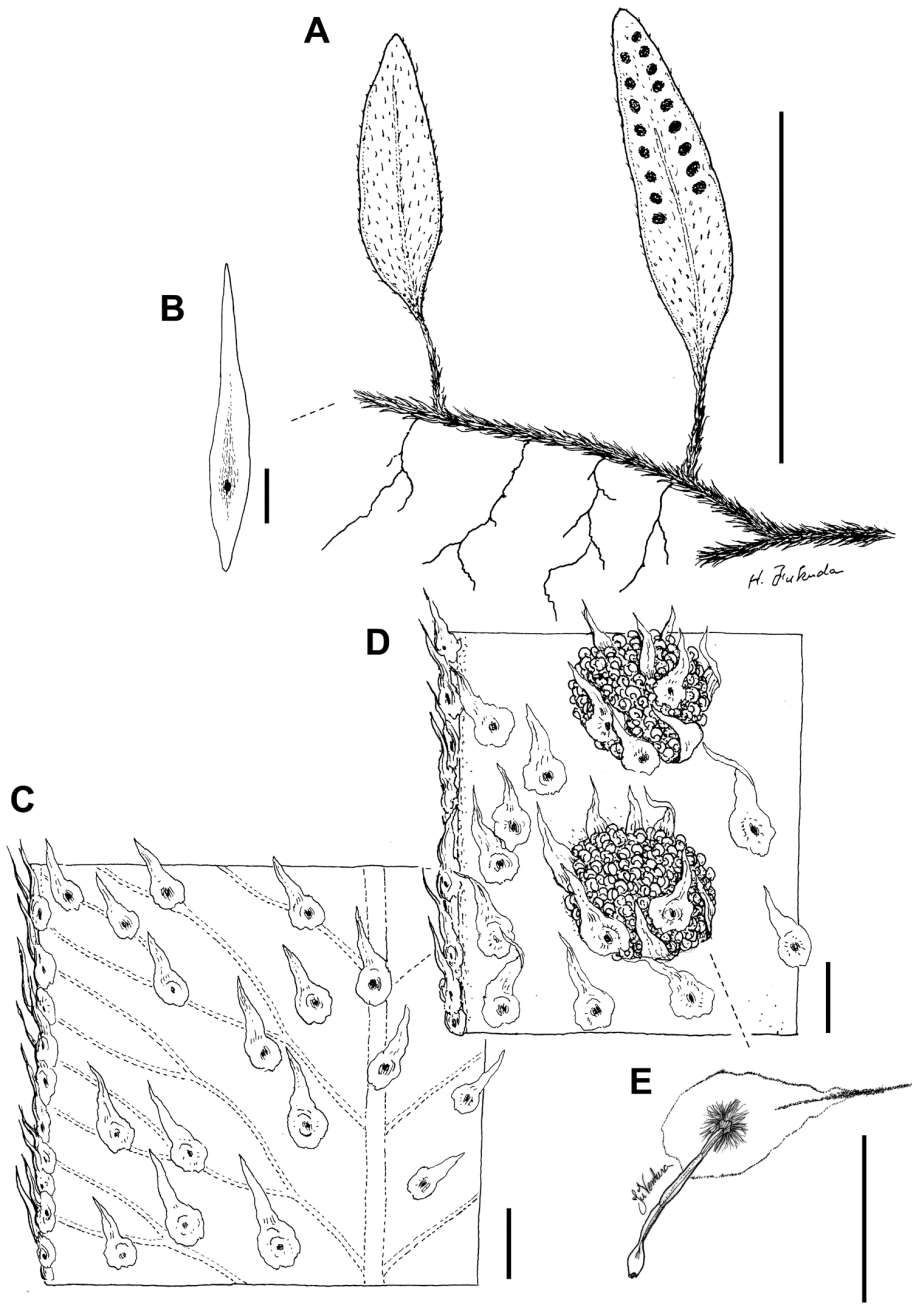
*Adetogramma chrysolepis* (Hook.) T.E.Almeida. **A** Habit **B** Rhizome scale **C** Sterile lamina detail showing free venation and laminar scales **D** Fertile lamina detail showing sori, paraphyses, and laminar scales **E** Paraphysis detail. Drawings A–D by H. Fukuda from *Dorr* et al. *6764* (NY); drawing E by Juliana Ventura from *T.E. Almeida & L.L. Giacomin 3121* (BHCB). Scale bars: **A** = 5 cm, **B–E** = 1 mm.

**Figure 3.**
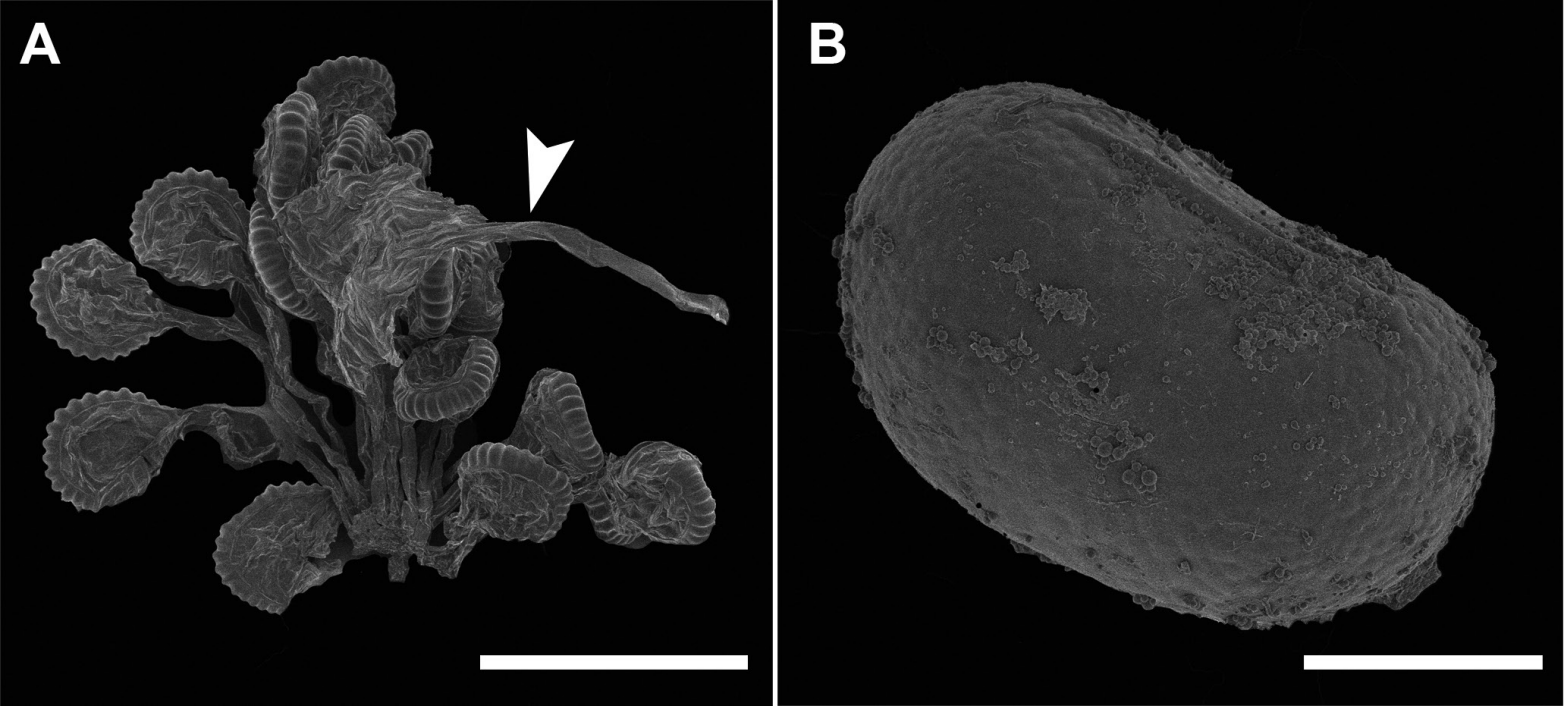
*Adetogramma chrysolepis* (Hook.) T.E.Almeida. **A, B** Scanning electron micrography (SEM); magnification 75× and 1750×, respectively **A** Sori showing sporangia and paraphysis (the latter indicated by an arrow head) **B** Spores. Images from *Almeida & Giacomin 3121* (BHCB). Scale bars: **A** = 500 μm, **B** = 20 μm.

In our analyses, *Serpocaulon*, a group segregated from *Polypodium* (Smith et al. 2006a), appears as sister to *P. chrysolepis* (0.97 PP, 88% BS), supporting the results of Schneider et al. (2004). Morphologically *Polypodium chrysolepis* can be readily distinguished from *Serpocaulon* by having free veins, non-clathrate scales, peltate scales covering the laminar surfaces, and peltate scales as paraphyses. These characters contrast with features of *Serpocaulon* species: regular anastomosing veins (goniophlebioid venation), clathrate scales, and the absence of scales in veins or laminar surface between veins or paraphyses, or if paraphyses are present, they are 2-3 celled glands (Smith et al. 2006a) (Table 2). Hernández et al. (2014) reported similarities in anatomical features of root cortical cells between *P. chrysolepis* and *Serpocaulon gilliesii* (C.Chr.) A.R.Sm, the only *Serpocaulon* species sampled in their study. Of all *Serpocaulon* species, *S. levigatum* (Cav.) A.R.Sm. is the only one that has entire leaves, and therefore the single species resembling *P. chrysolepis* morphologically. However, in addition to the characteristics mentioned above, *S. levigatum* differs from *P. chrysolepis* by having multiple rows of sori on each side of midvein and by rhizomes being covered by whitish wax-like deposits, with few roundish scales, not covering the entire surface of the rhizome (Smith et al. 2006a, Labiak and Prado 2008), while in *P. chrysolepis* only one row of sori is found on each side of midvein, and rhizomes lack whitish wax-like deposits and are covered by lanceolate rhizome scales (Figs 2, 3). Smith et al. (2006a) highlighted a possible trend of increasing pinnation in *Serpocaulon*, but the fact their results showed *S. levigatum* to be closely related to species with completely free, non-adnate pinnae would make the entire lamina shared with *P. chrysolepis* a homoplastic feature.

**Table 2.** Comparison of character states among *Adetogramma* and the morphologically or phylogenetically closest genera/groups.

	***Adetogramma***	***Serpocaulon*^1^**	**Grammitids**	***Pecluma***	***Polypodium*^2^**	***Pleopeltis*^3^**	***Microgramma*^4^**
**Rhizome**	Long-creeping, branched	Long- to short-creeping, sparingly branched	Short-creeping to erect, usually unbranched	Long- to short-creeping, unbranched	Long- to short-creeping, branched	Long- to short-creeping, branched	Long-creeping, branched
**Rhizome scales**	Peltate, non-clathrate	Peltate, clathrate	Basifixed, non-clathrate or clathrate, or absent	Basifixed to peltate, non-clathrate	Peltate, non-clathrate	Peltate, non-clathrate, to clathrate at margins	Peltate, non-clathrate
**Fronds**	Monomorphic	Monomorphic	Monomorphic, or the distal fertile portion modified	Monomorphic	Monomorphic	Monomorphic to dimorphic	Monomorphic to dimorphic
**Lamina**	Simple	Pinnatifid to pinnate, rarely simple	Simple to 3-pinnate	Pinnatisect to pinnate	Deeply pinnatifid to pinnate	Simple to pinnatifid, rarely pinnate-pinnatifid or more divided	Simple to lobate
**Indument on lamina**	Scales	Glabrous, trichomes, or scales (confined to costae and rachises)	Trichomes, and sometimes glands	Trichomes	Glabrous or with trichomes	Scales	Glabrous, trichomes and/or scales
**Veins**	Free	Regularly anastomosing (goniophlebioid), areoles with one included veinlet	Usually free, sometimes anastomosing with or without included free veinlets	Free, rarely anastomosing, but never reticulate	Free to anastomosing, with one single included veinlet	Free to anastomosing, areoles with 1-3 free or netted included veins	Anastomosing, with simple included veinlets
**Sori**	Round to oblong, 1 row between costa and margins	Round, 1-10 rows between costa and margins	Round to elongate, 1 row between costa and margins, or confluent	Round to oblong, 1 row between costa and margins	Round to oblong, 1-5 rows between costa and margins	Round to oblong, or linear, rarely marginal and coalescing, in 1 row between costa and margins	Round to elongate, 1 row between costa and margins, confluent or forming several irregular rows between costa and margins
**Paraphyses**	Peltate, pedicellate, scales	Absent or short 2-3-celled glands	Present or absent	Present, glandular trichomes	Absent or if present, filamentous or branched	Absent, or round peltate,	Trichomes or sessile scales, or absent
**Spores**	Bilateral, monolete, verrucate	Bilateral, monolete, verrucate, generally tuberculate, occasionally winged	Tetrahedral-globose, trilete	Bilateral, monolete, smooth to tuberculate	Bilateral, monolete	Bilateral, monolete, shallowly to prominently verrucate	Bilateral, monolete, tuberculate

^1^*Serpocaulon* circumscription follows Smith et al. (2006).

^2^*Polypodium* circumscription accepted here includes *Polypodium plesiosorum* and *Polypodium colpodes* species groups (sensu Tejero-Díez 2005, Sigel et al. 2014), *Polypodium dulce* species group (sensu Moran 1995) and *Polypodium vulgare* species group (sensu Haufler et al. 1995).

^3^*Pleopeltis* circumscription accepted here follows Smith and Tejero-Díez (2014).

^4^*Microgramma* circumscription follows Salino et al. (2008).

Sanín (2014, 2015) described a species of *Serpocaulon* with free veins (*S. tayronae* D.Sanín), but no phylogenetic evidence is presented to support its placement in *Serpocaulon*. Sanín also stated that *S. eleuterophlebium* (Fée) A.R.Sm. and *S. patentissimum* (Mett.) A.R.Sm. have free veins, although Hensen (1990) describes their venation as regularly anastomosing in the taxonomic treatment of the group (treated as the *Polypodium loriceum*-complex). More evidence is needed to elucidate the generic position of *S. obscurinervium* and *S. tayronae*, as is the case with several other groups morphologically related to *Polypodium* (Tejero-Díez 2005, Assis et al. 2016).

*Polypodium chrysolepis* was combined by Crabbe (1967) in *Microgramma*, but it exhibits morphological characters distinct from that genus. It has free venation while *Microgramma*, as circumscribed by Almeida et al. (unpublished data) and Salino et al. (2008), has anastomosing veins on the sterile fronds. Moreover, *P. chrysolepis* has lanceolate, peltate, long-stalked paraphyses (Fig. 2E) while in *Microgramma*, paraphyses (if present) are hairlike or lanceolate and sessile, never stalked (Table 2). *Polypodium chrysolepis* resembles some *Pleopeltis* species in having entire laminae, long-creeping rhizomes, and peltate paraphyses. The main difference between *Pleopeltis* and *Polypodium chrysolepis* lies in the shape of the paraphyses – ovate-lanceolate in *P. chrysolepis* vs. roundish in *Pleopeltis*. Furthermore, species of *Pleopeltis* with entire blades always have anastomosing veins (Table 2).

Grammitid ferns, the lineage sister to the *Polypodium chrysolepis*+*Serpocaulon* clade, are a very distinct group of species inside the polygrammoid ferns. Once recognized as a separated family (Parris 1990) or a subfamily inside Polypodiaceae (Tryon and Tryon 1982), this lineage can be distinguished by usually tetrahedral chlorophyllous spores, sporangia stalk reduced to a single cell wide in the middle, absence of scales in the fronds, and free or occasionally anastomosing without free included veinlets (Parris 1990, Sundue et al. 2010). Although sharing the free veins with *Polypodium chrysolepis*, we can distinguish the latter from grammitids by the bilateral spores, and the presence of scales in the blades (Table 2).

*Polypodium* *s.s.*, following Tejero-Díez (2005), includes the species groups of *Polypodium plesiosorum*, *Polypodium colpodes*, *Polypodium dulce* (sensu Moran 1995) and *Polypodium vulgare* species group (sensu Haufler et al. 1995). Using this circumscription, *Polypodium* *s.s.* remains a polyphyletic assemblage of species, with species from the *Polypodium dulce* complex apparently closer to *Pecluma* than to *Polypodium* *s.s.* (Otto et al. 2009, Assis et al. 2016); in fact, some species in this group were moved to *Pecluma* (Assis et al. 2016). Further studies are needed to define a monophyletic *Polypodium*. Nevertheless, following this circumscription, *Polypodium* *s.s.* can be distinguished from *P. chrysolepis* by its deeply-pinnatifid to pinnate leaves with free (in the *Polypodium dulce* complex species) to anastomosing veins, with a single included veinlet in each areole. Indument is also a useful character for separating *P. chrysolepis*: *Polypodium* *s.s.* shows glabrous to pilose laminar surfaces and the paraphyses, when present, are filamentous or branched, while *P. chrysolepis* has scaly laminae and the paraphyses are pedicellate scales. Table 2 summarizes the features and main differences amongst the related genera.

Our results do not support the inclusion of *Polypodium chrysolepis* in any genus previously recognized, including *Microgramma*, *Pleopeltis*, or *Polypodium* *s.s.* Therefore, we consider this species as constituting a separated, isolated lineage inside the polygrammoid clade. Because the species also has a morphology distinct from that of all other known genera in Polypodiaceae, we believe it merits recognition as a genus, and is described below.

### Taxonomic treatment

#### Adetogramma

Taxon classificationPlantaePolypodialesPolypodiaceae

T.E.Almeida gen. nov.

urn:lsid:ipni.org:names:77161864-1

##### Note.

*Adetogramma* is similar to *Microgramma* and *Pleopeltis* in its epiphytic habit, long-creeping rhizomes and in having entire leaves with one row of sori on each side of the midrib, but differs from these genera by having free veins (vs. veins anastomosing in *Microgramma* and *Pleopeltis*) and peltate, pedicellate, lanceolate paraphyses (vs. hairlike or lanceolate and sessile paraphyses in *Microgramma*, and round, peltate, pedicellate paraphyses in *Pleopeltis*).

##### Type.

*Adetogramma chrysolepis* (Hook.) T.E.Almeida, comb. nov., *Polypodium chrysolepis* Hook., Icon. Pl. 8: t. 721. 1845.

#### Adetogramma chrysolepis

Taxon classificationPlantaePolypodialesPolypodiaceae

(Hook.) T.E.Almeida comb. nov.

urn:lsid:ipni.org:names:77161865-1

Figures 2 , 3 , 4

Lepicystis chrysolepis (Hook.) Diels, Nat. Pflanzenfam. 1(4): 322, f. 167A–B. 1899. Type: Based on Polypodium chrysolepis Hook.
Microgramma chrysolepis (Hook.) Crabbe, Brit. Fern Gaz. 9: 316. 1967. Type: Based on Polypodium chrysolepis Hook.
Polypodium bangii Baker, Bull. Misc. Inform. Kew 1901: 145. 1901. Type: Bolivia. Yungas, 1890, *A.M. Bang 734* (lectotype, designated here: BM! [BM000936895]; isolectotypes: B! [B200075587], BR!, GH!, K! [K000590773], LE!, MO! [MO5472871)], NY! [NY00144786, NY00144787], US! [US00065725]).

##### Basionym.

*Polypodium chrysolepis* Hook., Icon. Pl. 8: t. 721. 1845.

##### Type.

Ecuador. Andes de Quito, *W. Jameson 37* (wrongly typed in protologue as “73”; lectotype, designated by Tryon et al., 1993, pg. 151: K! [K000590772]; isolectotypes: BM! [BM000936896], G!, FI [FI004543!]).

Plants epiphytic or epipetric, rarely terrestrial. Rhizomes long-creeping, branched, 0.6–0.9 mm wide, cylindrical, with four vascular bundles; short, perpendicular roots about 5–20 mm long, these regularly spaced, covered with brownish root hairs; rhizome scales 4.3–7.1 mm long, peltate, not clathrate, linear-lanceolate, with elongate cells, the margins entire from the base to the middle and toothed beyond the middle, with 1- or 2-celled marginal teeth, scales concolorous, stramineous and usually darker at the attachment point. Fronds remote, 2.2–4.5 mm apart, articulate, monomorphic. Stipes nearly absent to 55 mm long, 0.4–0.7 mm in diameter, covered with sparse peltate, lanceolate, sessile, non-clathrate, concolorous, stramineous scales, these 1.4–3.5 mm long, darker at the attachment point; phyllopodia darker than stipes. Laminae light green, 5.0–17.0 × 1.0–2.5 cm, simple, chartaceous, linear-lanceate to lanceolate, bases acuminate to attenuate, decurrent in the distal third of the stipe, apices acute to obtuse, laminar surfaces squamose on both sides, scales lanceolate, peltate, sessile, non-clathrate, concolorous, translucent, stramineous, darker at attachment point, slightly erose at bases and entire at apices, scales present also on abaxial and adaxial sides of costae and veins, and on the laminar margins, 1.3–2.6 mm long. Veins free, immersed, obscure, 1–2 furcate, not reaching laminar margins, midribs and lateral veins immersed on both sides of the laminae, not evident. Sori superficial, rounded to oblong, 1.6–2.6 × 1.9–5.0 mm, terminal to subterminal on veins, receptacles elongate, sporangia long-stalked, paraphyses present, scale-like, similar those of the laminar surfaces, peltate, pedicellate, with pedicels as long as those of the sporangia, paraphyses completely covering immature sori. Spores yellow, with verrucate surfaces.

##### Distribution and ecology.

Restricted to central and southern Andes, with known collections from Ecuador, Peru, Bolivia, and Argentina (Fig. 5). It occurs preferably in high elevation formations, ranging from 1,800 to 4,100 meters above sea level, with most collections between 3,000–4,000 m in the central part of the Andes, but found at lower elevations further south in Southern Bolivia and Argentina. *Adetogramma chrysolepis* is mostly epiphytic or epipetric, rarely terrestrial. Epiphytic specimens usually grow on *Polylepis*, *Berberis*, or *Buddleja*, inside highland humid montane forests (Yungas). Epipetric ones were recorded as growing on rocks inside Yungas forest or in sub-alpine grasslands with scattered shrubs, normally associated with mosses (de la Sota 1960). According to herbarium sheet labels, the species also occurs in secondary forests and modified areas.

##### Conservation status.

Least Concern (LC - IUCN 2016). *Adetogramma chrysolepis* presents EOO of 1,108,559.773 km^2^ and AOO of 5,300 km^2^, respectively, and its conservation status is considered Least Concern. However, the species occurs in a fragile environment that is undergoing an increasing pressure due to human settlement, and extensive grazing by cattle and camelids. Although it is known from at least 40 localities and occurs in several protected areas (e.g., Parque Nacional Carrasco, Cochabamba, Bolivia; Reserva Nacional de Flora e Fauna de Tariquía, Tarija, Bolivia; Parque Nacional Huascarán, Ancash, Peru; Santuario Nacional de Ampay, Apurimac, Peru; Parque Nacional del Rio Abiseo, San Martín, Peru) no information on population fluctuations is available. Decline in the quality and area of occupancy in the near future seems feasible and it is advised that the species be monitored.

##### Etymology.

The generic epithet refers to the most distinctive character of the species, the free venation (Fig. 2C); from the Greek adetos-, free and -gramma, line.

##### Notes.

*Adetogramma* is a monotypic genus, and although its sole species, *A. chrysolepis*, varies in laminar size and shape and stipe length, all other characters, such as the rhizome and stipe scales, venation, and paraphyses, are constant. Specimens from the Argentinean Provinces of Tucumán and Salta and Bolivian Provinces of Tarija and Chuquisaca have very long, linear laminae, and longer stipes, but in other characters are congruent with the circumscription here adopted for *A. chrysolepis*. This variation may reflect colonization of drier, seasonal habitats in a subtropical region.

**Figure 4.**
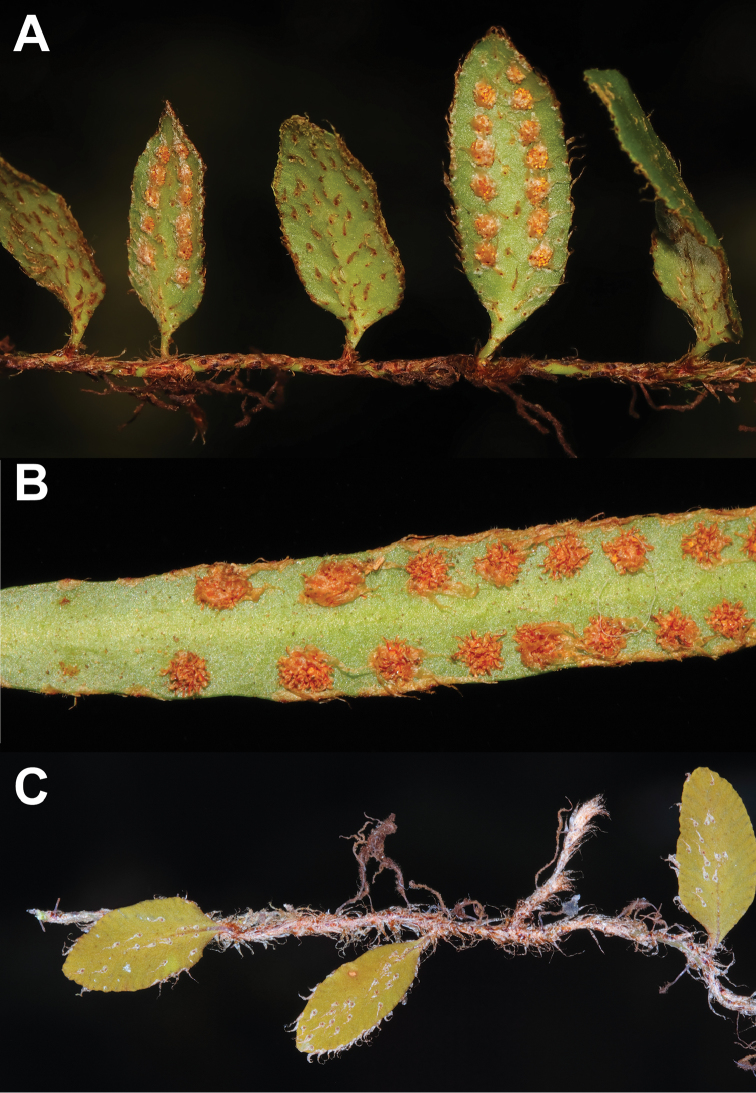
*Adetogramma chrysolepis* (Hook.) T.E.Almeida. **A** Fertile and sterile fronds **B** Detail of abaxial surface of fertile frond, showing laminar scales and paraphyses **C** Juvenile sterile leaves. (A) and (B) from *C. Martín 730* (SI), (C) from *C. Martín 479* (SI).

Morphologically, *Adetogramma chrysolepis* shares features with several neotropical genera of Polypodiaceae (Table 2), while presenting unique characteristics within the group. Free veins (Figs 2C, 4C) are shared with the *Polypodium dulce* species group, some *Pecluma* species, a few *Pleopeltis* species, few pinnatisected species of *Serpocaulon*, *S. tayrona* and most grammitids. Presence of peltate scales on the receptacle is shared between *Adetogramma* and *Pleopeltis* (Figs 2D–E, 3A, 4A–B). In both genera, paraphyses may almost completely cover the sporangia in immature sori, and presumably have a protective function.

Kreier et al. (2008) hypothesized that *Serpocaulon*, sister group to *Adetogramma*, has the Bolivian Andes and adjacent southeastern Brazil as its ancestral area. According to this hypothesis, the Bolivian Andes formed a path for migration and successful colonization of the Northern Andes, with subsequent migration into Central America and Caribbean regions. With *Adetogramma* as sister to *Serpocaulon*, we believe the hypothesis of the Bolivian Andes as putative ancestral area of the *Adetogramma*+*Serpocaulon* clade common ancestor to be more likely. From Bolivia, *Adetogramma* could have dispersed southwards where colder climate allowed it to colonize lower elevation habitats, and also migrated northwards to Peru and Ecuador. *Adetogramma chrysolepis* may represent a single relictual species from a previously more diverse and geographically widespread group, or a single, undiversified lineage that colonized high elevation environments. More detailed phylogenetic or phylogeographic studies are required to support or refute these hypotheses.

##### Specimens examined.

**ARGENTINA. Jujuy**: San Antonio, 27 Apr 2015, *C. Martín 479* (SI); Valle Grande, 14 Apr 2016, *C. Martín 730* (SI). **Salta**: Salta, 21 Nov 1945, *S.A. Pieroffi* 1326 (NY!); Santa Victoria, 05 Dec 2015, *C. Martín 673* (SI). Tucumán: s.l., 1952, *H. Brücher s.n.* (LP!); s.l., s.d., *L. Castillón 2248* (BM!); s.l., s.d., *M. Lillo 11534* (BM!, GH!, K!, NY!); La Ventanita, s.d., *M. Lillo 16713* (BM!, GH!, K!); Burruyacu, 20 Jan 1947, *Borsini s.n.* (LP!); Chicligasta, 11 Dec 1925, *S. Venturi 4062* (BM!, GH!); idem, 13 Feb 1924, *S. Venturi 3151* (GH!); Tafi del Vale, Jan 1912, *L. Castillón 35* (BM!, GH!, K!, LP!, NY!). **BOLIVIA**. **Chuquisaca**: Hernando Siles, 09 Nov 2007, *J. Villalobos 928* (MO!, UC). **Cochabamba**: Ayopaya, 07 May 1990, *E. Hennipman 8148* (LPB!); idem, 28 Oct 2007, *J. Terán 1486* (MO!); idem, 30 Oct 2007, *J. Terán 1563* (MO!); Carrasco, 20 Mar 1991, *I. Hensen 1819* (LPB!); idem, 25 Feb 1996, *M. Mercado 501* (MO!); Chapare, 30 Sep 2001, *J.R.I. Wood 17271* (LPB!); Cochabamba, 27 Jan 1950, *W.M.A. Brooke 6081* (BM!, NY!); Jose Carrasco Torrico, 27 Jun 1996, *M. Kessler 6763* (NY!, UC); idem, 02 Jul 1996, *M. Kessler 6877* (NY!, UC); Tiraque, 10 May 2005, *E. Zurita 390* (MO!). **La Paz**: Bautista Saavedra, 27 Apr 1993, *P. Gutte 559* (LPB!); Franz Tamayo, 24 Feb 2008, *A.F. Fuentes Claros 11982* (MO!, UC); idem, 04 Mar 1980, *J. Krach 9207* (LPB!); idem, 06 Apr 2009, *M.I.L. Rivera 599* (MO!); Inquisivi, 21 Dec 1989, *L.J. Dorr 6764* (LPB!, NY!); idem, 18 Feb 1989, *M.A. Lewis 35242* (LPB!, MO!, NY!, UC); idem, 09 Mar 1991, *M.A. Lewis 38230* (MO!); idem, 04 Sep 1991, *M.A. Lewis 39744* (F, GH!, LPB!, MO!); Larecaja, 1818, *G. Mandon 1560* (BM!, G!, K!, NY!); Murillo, 16 May 1985, *J.C. Solomon 13742* (LP!, LPB!, MO!, NY!); Nor Yungas, XI/1900, *O. Buchtien 2751* (P!); idem, 07 Mar 1969, *H. Doppelbaur s.n.* (MO!); Pedro Domingo Murillo, 17 Mar 2012, *T.E. Almeida 3121* (BHCB!, LPB!); Pongo, s.d., *W.M.A. Brooke 5456* (BM!, NY!); Unduavi, 1890, *A.M. Bang 734* (B!, BR!, GH!, K!, LE!, MO!, US!); idem, 30 Mar 1977, *J.D. Boeke 1375* (NY!); idem, Feb 1914, *O. Buchtien 420* (B!, BM!, G!, GH!, K!, NY!, P!); idem, 19 Jun 1912, *E. Rosenstock 49* (B!, P!). **Santa Cruz**: Manuel M. Caballero, 11 Apr 2004, *R. Nuñez Cabrera 682* (MO!, NY!, UC, USZ!); idem, 08 Mar 2012, *T.E. Almeida 3083* (BHCB!, HSTM!, LPB!); s.l., 26 Oct 1928, *J. Steinbach 8531* (BM!, GH!, K!, MO!, NY!). **Tarija**: Aniceto Arce Ruiz, 13 Jun 2004, *I. Jimenez 2420* (NY!, UC); Arce, 10 Nov 2004, *H. Huaylla 1509* (MO!); idem, 27 Jun 2005, *H. Huaylla 1882* (MO!); idem, 12 Nov 2004, *M. Serrano 5082* (MO!); idem, 12 Nov 2004, *M. Serrano 5136* (MO!); idem, 03 Feb 2005, *M. Serrano 5945* (MO!); O’Connor, 22 Oct 1983, *S.G. Beck 9651* (LPB!); s.l, s.d., *M. Cárdenas 1036* (GH!); s.l., 07 Mar 1969, *H. Doppelbaur 291* (M!); s.l., 06 May 1928, [illegible]*1569* (B!); s.l., 21 Aug 1926, [illegible] *2559* (B!). **ECUADOR**. **Pichincha**: Quito, s.d., *W. Jameson 73* (G!, K!). **PERU**. **Amazonas**: s.l., 03 Aug 1962, *J.J. Wurdack 1592* (GH!). **Ancash**: Carhuaz, 14 Feb 1985, *D.N. Smith 9571* (MO!, USM); idem, 18 Jul 1985, *D.N. Smith 11264* (BM!, ENAG, F, GH!, MICH, MO!, NY, UC, USM); Huari, 08 May 1986, *D.N. Smith 12415* (BM!, F, LPB!, MO!, NY, UC); idem, 13 Jun 1986, *Smith 12648* (MO!); Yungay, 13 Jan 1985, *Smith 9150* (GH!, MO!, USM); idem, 17 Apr 1985, *D.N. Smith 10326* (MO!, USM); idem, 19 Apr 1985, *D.N. Smith 10463* (MO!, USM). **Apurimac**: Abancay region, Oct 1935, *students of Santander C. s.n.* (UC); Abancay, Tamburco, 6 Jun 2015, *V. Zuñiga 452* (USM). **Cusco**: s.l., 31 May 2002, *W.L. Galiano 4034* (MO!); s.l., 14 Feb 1987, *P.Núñez Vargas 6988* (MO!); s.l., 10 Jun 2002, *L.Valenzuela Gamarra 238* (NY!, MO!); La Convención, 11 Jul 2003, *E. Bonino 832* (MO!); idem, 23 Jul 2003, *E. Bonino 909* (MO!, UC); idem, 26 Mar 2004, I. *Huamantupa 4428* (MO!); idem, 21 Sep 2005, *I. Huamantupa 6844* (MO!, USM!); idem, 30 Mar 1939, *C. Vargas 4505* (MO!); Urubamba, 23 Apr 1982, *B. Peyton 51* (MO!); idem, 04 Jul 1982, *B. Peyton 766* (MO!); idem, 17 Aug 1982, *B. Peyton 1053* (GH!, MO!); idem, 05 Nov 1988, *A. Tupayachi 758* (GH!, MO!, NY!); idem, 30 Dec 1963, *L. Valenzuela 14983* (GH!). **Huánuco**: Yanano, 13-16 May 1923 *J.F. Macbride 3826* (G!). **La Libertad**: Pataz, 24 Feb 1986, *K. Young 2988* (NY); s.l., 23 Jun 1974, *A. López M. 8129* (G!, MO!, NY!). **Lambayeque**: Ferreñafe, 9 Jun 2012, *L. García Llatas s.n.* (USM). **San Martín**: Mariscal Cáceres, Huicungo, 15 Jun 2001, *B. León 5249* (NY!, USM).

**Figure 5.**
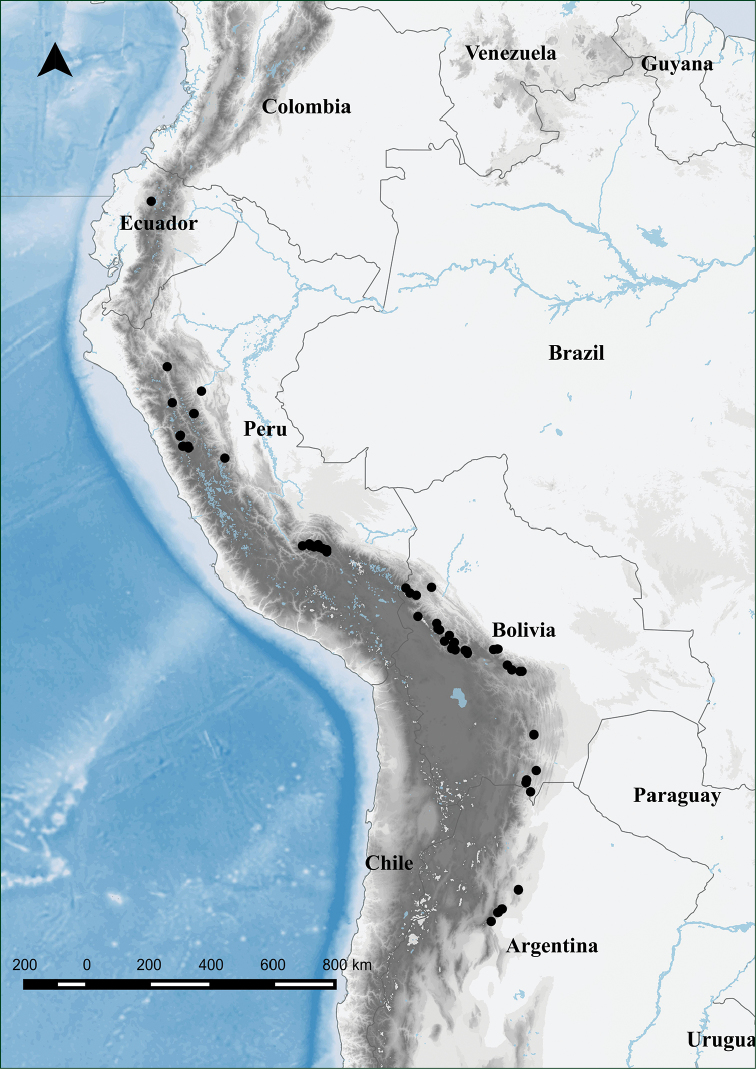
Distribution of *Adetogramma chrysolepis*.

## Supplementary Material

XML Treatment for Adetogramma

XML Treatment for Adetogramma chrysolepis

## Appendix

**Table:** Collection information for voucher specimens and GenBank accession numbers for sequences used in this study. Locality and voucher specimen (herbarium) are given to sequences newly generated in this study. *sequences submitted to GenBank

Taxon	*rbc*L	*rps*4-*trn*S
*Adenophorus oahuensis* (Copel.) L.E.Bishop	AY057382	AY096236
*Adetogramma chrysolepis* (Hook.) T.E.Almeida; Bolivia, *Almeida 3083* (BHCB)	KY847865*	KY847858*
*Adetogramma chrysolepis* (Hook.) T.E.Almeida; Bolivia, *Almeida 3121* (BHCB)	KY847859*	KY847863*
*Aglaomorpha pilosa* (J.Sm.) Copel.	AY529156	AY529180
*Alansmia cultrata* (Willd.) Moguel & M.Kessler	GU376496	JN654943
*Arthromeris himalayensis* (Hook.) Ching	JQ685378	JQ685442
*Campyloneurum gracile* A.Rojas; Panama, *Salino 15357* (BHCB)	KY847860*	KY847864*
*Campyloneurum phyllitidis* (L.) C.Presl	KT780752	KT794132
*Davallia solida* (G.Forst.) Sw.	AY096193	AY096210
*Dictymia brownii* (Wikstr.) Copel.	DQ227292	DQ227295
*Goniophlebium percussum* (Cav.) W.H.Wagner & Grether	AY362561	AY362628
*Gymnogrammitis dareiformis* (Hook.) Tardieu & C.Chr.	AY096201	JQ685456
*Lecanopteris carnosa* (Reinw.) Blume	AF470322	AY096227
*Lellingeria apiculata* (Klotzsch) A.R.Sm. & R.C.Moran	GU387021	GU387046
*Lellingeria brevistipes* (Kuhn) A.R.Sm. & R.C.Moran	GU387030	GU387049
*Lemmaphyllum carnosum* (Hook.) C.Presl	GU126698	GU126717
*Lepidomicrosorium subhemionitideum* (Christ) P.S.Wang	GU126693	GU126711
*Lepisorus heterolepis* (Rosenst.) Ching	GQ256270	GQ256344
*Leptochilus digitatus* (Baker) Noot.	JX103695	EU363250
*Leucotrichum madagascariense* Rakotondr. & Rouhan	JN654924	JN654949
*Leucotrichum organense* (Gardner) Labiak	GU376490	JN654946
*Loxogramme salicifolia* (Makino) Makino	DQ227294	DQ227297
*Melpomene flabelliformis* (Poir.) A.R.Sm. & R.C.Moran	GU387028	GU387114
*Melpomene melanosticta* (Kunze) A.R.Sm. & R.C.Moran	GU387024	GU387115
*Microgramma baldwinii* Brade; Brazil, *Almeida* 2631 (BHCB)	KY847861*	KY847866*
*Microgramma bifrons* (Hook.) Lellinger	AY362582	AY362654
*Microgramma mauritiana* (Willd.) Tardieu	DQ642148	DQ642185
*Microgramma persicariifolia* (Schrad.) C.Presl	KT780753	KT794133
*Microsorum membranaceum* (D.Don) Ching	EU482963	AY725047
*Mycopteris attenuatissima* (Copel.) Sundue	GU476927	GU387121
*Mycopteris subtilis* (Klotzsch) Sundue	GU476875	GU387128
*Neocheiropteris palmatopedata* (Baker) Christ	JX103706	GQ256396
*Niphidium crassifolium* (L.) Lellingerl; Brazil, *Almeida 3247* (BHCB)	KY847862*	KY847867*
*Niphidium nidulare* (Rosenst.) Lellinger	EF551064	EF551080
*Oleandra pistillaris* (Sw.) C.Chr	AB232405	AY096209
*Pecluma ptilotos* (Kunze) M.G.Price	AY362588	AY362661
*Phlebodium pseudoaureum* (Cav.) Lellinger	AY362589	AY362663
*Platycerium andinum* Baker	DQ164446	DQ164477
*Pleopeltis angusta* Willd.	EU650122	EU650161
*Pleopeltis desvauxii* (Klotzsch) Salino	AY362584	AY362657
*Polypodium vulgare* L.	JF832081	EF551081
*Pyrrosia angustata* (Sw.) Ching	DQ642165	DQ642204
*Selliguea plantaginea* Brack.	EF463262	EU128510
*Serpocaulon articulatum* (C.Presl) Schwartsb. & A.R.Sm.	DQ151910	DQ151935
*Serpocaulon dissimile* (L.) A.R.Sm.	DQ151908	DQ151933
*Serpocaulon levigatum* (Cav.) A.R.Sm.	DQ151917	EF551103
*Serpocaulon loriceum* (L.) A.R.Sm.	EF551074	EF551104
*Stenogrammitis hellwigii* (Mickel & Beitel) Labiak	GU386990	GU387058
*Stenogrammitis hildebrandtii* (Hieron.) Labiak	GU386975	GU387059
*Synammia feuillei* (Bertero) Copel.	AY362597	AY362670
